# Roles of autophagy-related genes in the therapeutic effects of Xuanfei Pingchuan capsules on chronic obstructive pulmonary disease based on transcriptome sequencing analysis

**DOI:** 10.3389/fphar.2023.1123882

**Published:** 2023-05-18

**Authors:** Ye Zhang, Xiaoming Xue, Lihong Meng, Dian Li, Wenxiao Qiao, Jinyun Wang, Di Xie

**Affiliations:** Graduate School, Shanxi University of Chinese Medicine, Taiyuan, China

**Keywords:** chronic obstructive pulmonary disease, Xuanfei Pingchuan capsule, autophagy, transcriptional group sequencing, MAPK pathway

## Abstract

**Objective:** Autophagy plays an important role in the occurrence and development of chronic obstructive pulmonary disease (COPD). We evaluated the effect of Xuanfei Pingchuan capsule (XFPC) on autophagy-related genes of COPD by a bioinformatics analysis and experimental verification.

**Methods:** The best treatment duration was screened by CCK8 assays. HBE cells were divided into three groups: blank, CSE and XFPC. After intervened by XFPC, HBE cells were collected and sent to Shenzhen Huada Gene Company for transcriptome sequencing. Subsequently, differential expression analyses, target gene prediction, and function enrichment analyses were carried out. Expression changes were verified in HBE cells by real-time Quantitative PCR (RT-qPCR) and western blotting (WB).

**Results:** The result of differential expression analysis displayed that 125 target genes of HBE cells were mainly related to mitogen-activated protein kinase (MKK) binding, interleukin 33 binding, 1-Pyrroline-5-carboxylate dehydrogenase activity, and the mitogen-activated protein kinase (MAPK) signal pathway. Among the target genes, the core genes related to autophagy obtained by maximum neighborhood component algorithm were *CSF1*, *AREG*, *MAPK9*, *MAP3K7*, and *AKT3*. RT-qPCR and WB methods were used to verify the result, it showed similar expression changes in *CSF1*, *MAPK9*, *MAP3K7*, and *AKT3* in bronchial epithelial cells to those in the bioinformatics analysis.

**Conclusion:** Through transcriptome sequencing and validation analysis, we predicted that CSF1, MAPK9, MAP3K7, and AKT3 may be the potential autophagy-related genes that play an important role in the pathogenesis of COPD. XFPC may regulate autophagy by down-regulating the expression of CSF1, MAPK9, MAP3K7, and AKT3, thus achieving the purpose of treating chronic obstructive pulmonary disease.

## 1 Introduction

Chronic obstructive pulmonary disease (COPD) is a major clinical inflammatory pulmonary disease caused by smoking and airway/alveolar abnormalities. It is characterized by persistent respiratory symptoms and airflow restriction, usually caused by long-term exposure to harmful particles or gases (Global et al., 2020). Acute exacerbation is an important event in the clinical process of COPD, and also an important factor affecting the health status and prognosis of patients. In recent years, traditional Chinese medicine has good clinical efficacy in preventing and treating COPD, and has achieved remarkable results (Li Jiansheng, 2020). Therefore, it is necessary to further clarify its mechanism.

The pathological mechanism of COPD is complex, involving inflammation, oxidative stress (Lee et al., 2019), protease-antiprotease imbalance (Brandsma et al., 2020), mitochondrial dysfunction (Agustí et al., 2019), apoptosis (Lan et al., 2019), and endothelial injury and repair imbalance (García-Lucio et al., 2018). Autophagy maintains cellular homeostasis mainly by the lysosome-dependent degradation of damaged proteins, lipids, and organelles (Nakamura et al., 2018). In the acute exacerbation of COPD, airway epithelial cells secrete a large number of inflammatory factors, and the level of autophagy in the lung tissue increases, resulting in the shortening and even death of the cilia length of airway epithelial cells, causing the obstruction of cilia movement function, resulting in airway mucus hypersecretion (Lv et al., 2020; Wang et al., 2018).

Xuanfei Pingchuan Capsule (XFPC) is a proprietary Chinese medicine preparation of Shanxi Traditional Chinese Medicine Hospital. Previous studies have shown that XFPC had obvious therapeutic effect on COPD, especially for patients with phlegm-heat accumulation syndrome (Xue et al., 2016a; Xue et al., 2016b). The results of vitro experimental studie have shown that XFPC could significantly inhibit autophagy and p38 phosphorylation in human bronchial epithelial (HBE) cells induced by cigarette smoke extract (CSE), thus playing a role in repairing the COPD cell model (Xue et al., 2021). However, its specific mechanism still needs further study.

At present, the mechanism of XFPC treating COPD by affecting autophagy level is not fully clarified. Next-generation sequencing technology and RNA sequencing can analyze transcriptome sequences, mine differentially expressed genes (DEGs), and even detect gene sequences of species without genome reference (YANG et al., 2022). In this study, we intends to conduct transcriptome sequencing and analysis on CSE-induced HBE cells, so as to find the target gene related to autophagy in HBE cells treated by XFPC, and clarify its mechanism.

## 2 Materials and methods

### 2.1 Sample processing

The ingredients of XFPC include roasted ephedra (10 g), almonds (10 g), Scutellaria (10 g), perillaseed (10 g), Platycodongrandiflorum (10 g), cortex mori radices (10 g), Aster (15 g), Flos Farfaraes (15 g), lepidium seed (10 g), Pinellia ternata (10 g), liquorice (licorice, 10 g), Schisandra (10 g), Ginkgobiloba (10 g), Angelica (15 g), *Astragalus* (30 g), and one pairof Gecko. XFPC were provided by the preparation room of ShanxiAcademy of Traditional Chinese Medicine (Taiyuan, China). Refer to Qizhen capsule (Guo et al., 2021) and Qiliqiangxin Capsule (Li et al., 2022) for the preparation method of liquid medicine. Bronchial epithelial HBE cells lines were purchased from the Shanghai Cell Bank.

HBE cells were divided into three groups, a control (Control), model (Model), and experimental (XFPC) group, with three replicates per group, there are nine samples in total. Drug powder from 23 XFPC capsules (approximately 11.5 g) was supplemented with 400 ml of distilled water, heated to boiling, filtered to obtain the supernatant, maintained at room temperature, and filtered to obtain the supernatant, yielding the XFPC medicine solution. Five cigarettes were burned completely under normal conditions with no wind. The completely burned cigarette smoke was collected in an inverted 50 ml centrifuge tube, 50 ml of complete medium was added, and smoke liquid was obtained. HBE cells were seeded in 3 (columns) × 2 (rows) 6-well plates with 2 ml of liquid in each well at 1.2 × 10^6^ cells per well. When the HBE cells in the 6-well plate were confluent, they were counted. Then, 1 ml of complete medium + 1 ml of smoke liquid was added to each well in the model and experimental groups. In the control group, 2 ml of complete medium was added to each well. After 24 h of modeling, the HBE cells were observed under a microscope. Then, the liquid was blotted and replaced with the XFPC prescription liquid at 1 ml of complete medium plus 1 ml of liquid per well. After culturing for 6 h, 12 h, and 24 h, HBE cells at the three time points were harvested and stored at -80°C for future use. Each group was designed with three experimental repetitions, and each repetition was designed with three holes.

### 2.2 Cell proliferation and toxicity assays to determine the optimal treatment duration

HBE cells were seeded in 96-well plates and divided into an experimental group and a control group. The blank group was not inoculated with HBE cells. In the experimental group, 10 μL of the drug solution was added to each well. In the control group and blank group, 10 μL of medium was added to each well. In the blank group, no treatment was administered. Each group was repeated three times. Finally, the HBE cells were cultured in a 37°C incubator. At 0, 6, 12, and 24 h after the intervention, the medium was replaced with medium containing 10% CCK-8, samples were placed in an incubator for 0.5 h, and the optical density D(λ) at a wavelength of 450 nm was measured using a microplate reader. Cell survival rate (%) = 100 × [(As-Ab)/(Ac-Ab)], and Inhibition rate (%) = 100 × [(Ac-As)/(Ac-Ab)], where As (experimental well) is the absorbance of the medium containing cells, CCK-8, and the drug to be tested; Ac (control well) is the absorbance of the culture medium containing cells, CCK-8, and no drug to be tested, and Ab (blank well) is the absorbance of the culture medium without cells and the drug to be tested, and CCK-8.

### 2.3 Transcriptome sequencing

HBE cells were stored on dry ice and sent to Shenzhen Huada Gene Company for transcriptome sequencing using Illumina technology. The raw sequencing data were filtered to remove reads with low quality (Reads with less than 15 bases and more than 20% of the total base number of the reads are defined as low-quality reads), adapters, and a highher than 5% content of unknown bases N) using SOAPnuke, independently developed by BGI. For samples with good quality and sufficient sequencing data, most of the transcripts will be completely covered, and the reads will be evenly distributed in all regions of the transcripts. HISAT was then used to compare the clean reads to the reference genome sequence. Bowtie2 was used to compare the clean reads to reference gene sequences, and RSEM was used to calculate gene expression levels in individual samples. The species name for the reference genome was *Homo sapiens*, the source was NCBI, and the version was *GCF_000001405_GRCh37. p13*.

### 2.4 Bioinformatics analysis

Referring to the differential gene detection method based on sequencing published by Audic S. et al. On Genome Research (Audic et al., 1997), BGI has developed a strict algorithm to screen the differentially expressed genes between the two samples. The threshold values for significant differential expression were a fold change value greater than 2 (i.e., |log_2_FC| > 1) and *p* < 0.05. Then, we make multiple hypothesis tests to correct the *p*-value of the difference test, and determine the domain value of *p*-value by controlling the FDR (False Discovery Rate). Gene Ontology (GO) biological process and Kyoto Gene and Genome (KEGG) pathway enrichment analyses were carried out to infer the biological functions of the predicted target genes. Enrichment analyses were performed using the phyper function in R to obtain *p*-values, and Q-values were obtained by the FDR correction of *p*-values. Functions with Q ≤ 0.05 were regarded as significantly enriched. The CytoHubba plug-in was used to calculate the genes with high node scores using the maximum neighborhood component (MNC) algorithm and to identify core genes (Hub-Gene) with a high degree of node centrality.

### 2.5 qRT-PCR validation of gene expression

RNA in HBE cells was extracted using the TRIgent Kit (Mei5 Biotechnology Co.,Ltd.). The integrity of the total RNA was determined by 1% agarose gel electrophoresis, and the content and purity were detected by an ultraviolet spectrophotometer. cDNA synthesis was carried out in a 20 μL system on ice according to the reagent instructions, incubated at 50°C for 5 min, and heated at 85°C for 5 s to inactivate the enzyme. The amplification reaction system was configured in strict accordance with the kit instructions, and the qPCR process was completed on a real-time fluorescent quantitative PCR instrument. The amplification reaction programs were 95°C for 60 s, 95°C for 15 s, 65°C for 15 s, and 72°C for 30 s, repeated 40 times. Relative expression levels were obtained by the 2^−ΔΔCT^ method. The qPCR primers were designed by software Primer 5.0, as shown in Table 1. The internal reference gene was *ß*-actin, whose upstream sequence was *CAT​GTA​CGT​TGC​TAT​CCA​GGC* and downstream sequence was *CTC​CTT​AAT​GTC​ACG​CAC​GAT*. The primers were synthesized by Kingsley Biotechnology Co., Ltd., and all primers were detected by PCR.

**TABLE 1:** Specific primers used for qRT-PCR amplification.

Gene	Forward primer (5’→3′)	Reverse primer (5’→3′)	Length of PCR product/bp
CSF1	AGA​CCT​CGT​GCC​AAA​TTA​CAT​T	AGG​TGT​CTC​ATA​GAA​AGT​TCG​GA	248
AREG	GAG​CCG​ACT​ATG​ACT​ACT​CAG​A	TCA​CTT​TCC​GTC​TTG​TTT​TGG​G	121
MAPK9	GAA​ACT​AAG​CCG​TCC​TTT​TCA​GA	TCC​AGC​TCC​ATG​TGA​ATA​ACC​T	186
MAP3K7	ATT​GTA​GAG​CTT​CGG​CAG​TTA​TC	CTG​TAA​ACA​CCA​ACT​CAT​TGC​G	186
AKT3	TGA​AGT​GGC​ACA​CAC​TCT​AAC​T	CCG​CTC​TCT​CGA​CAA​ATG​GA	160

### 2.6 Western blot detection of related proteins

HBE cells samples were supplemented with 250 μL of lysis solution, shaken, and mixed until complete lysis. The lysed samples were centrifuged at 12,000 × *g* at 4°C for 15 min, and the supernatant was collected for protein quantification and stored in a -80°C refrigerator. PAGE was performed using different gel concentrations according to the molecular weight of the target protein (i.e., using a low-concentration gel for high-molecular-weight proteins and a high-concentration gel for low-molecular-weight proteins). Then, 20 μg of total protein was added to each comb well and an appropriate amount of electrophoresis buffer was added for electrophoresis for 90 min. The samples were transferred to a PVDF membrane and stained with Ponceau to check whether the membrane transfer was successful. Samples were blocked with 5% skim milk powder for 1 h at room temperature or overnight at 4°C. The primary antibodies were diluted according to the manufacturers’ instructions, added to the blocking solution, and diluted to the desired concentration. Samples were incubated for 2 h at room temperature or overnight at 4°C. Samples were washed with TBST. The HRP-labeled secondary antibody was diluted at 1:1,000 and incubated with the membrane at 37°C for 1 h, followed by washing with TBST and the application of ECL light to the front of the membrane for 5 min in the dark. After the film was exposed, it was developed for 2 min and then fixed.

### 2.7 Statistical analysis

SPSS 26.0 was used for analyses. For data following the normal distribution, the results are expressed as the mean ± standard deviation ( $\bar{x}$ ±*s*), and comparisons among the three groups were performed by analysis of variance. Data with a skewed distribution are expressed as the median value (interquartile range) [M(Qr)], and comparisons between groups were performed by the Mann–Whitney *U* test. The count data are expressed as n (%), and comparisons were performed by the Chi-squared test or Fisher’s exact test. A statistically significant difference was defined as *p* < 0.05.

## 3 Results

### 3.1 Cell proliferation and toxicity assays

**FIGURE 1:**
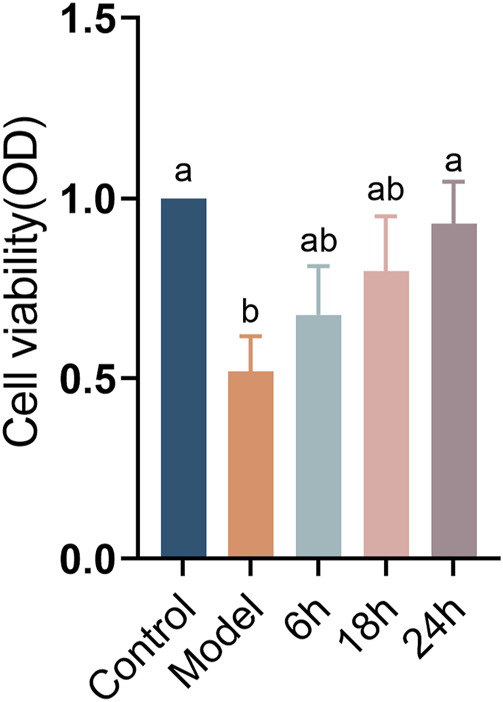
Effect of different administration times on viability in an HBE cell model of COPD.

As shown in Figure 1, setting the cell survival rate in the blank group to 100%, survival in the blank model group was 51.97%, supporting the establishment of the model of CSE-induced COPD. The cell survival rates were 67.68%, 79.83%, and 92.96% for XFPC administration times of 6 h, 12 h, and 24 h, respectively. Therefore, 24 h of administration was selected for follow-up analyses.

### 3.2 Sequencing data and gene annotation

**FIGURE 2:**
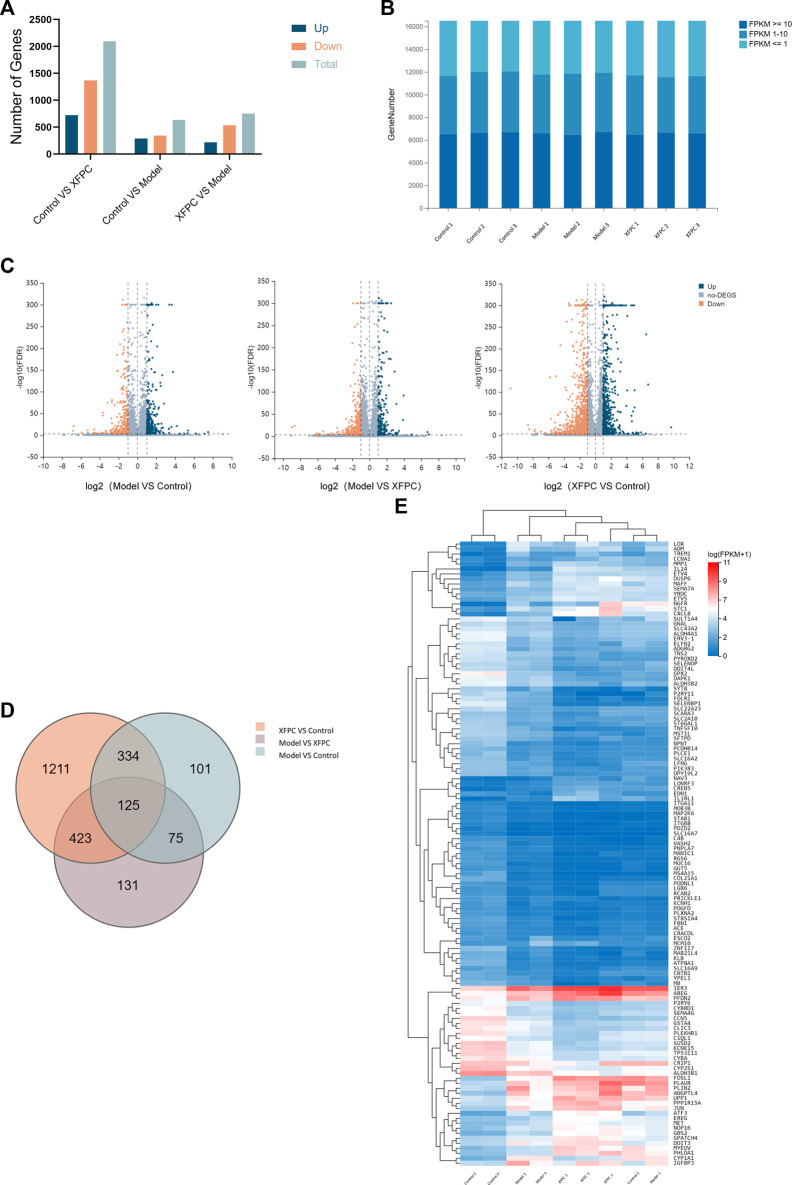
**(A)** Summary of differentially expressed genes (DEGs). **(B)** Prediction of target genes; 109 target genes were obtained from the intersection of DEGs among the control group, model group, and XFPC group. **(C)** Volcano plot; orange indicates downregulation and blue indicates upregulation.

The average output for nine sample detected by the BGISEQ platform was 6.68 Gb. The average alignment rate of reads for each sample to genomes was 94.66%, and the average alignment rate of gene sets was 61.36%. A total of 16,507 genes were detected. There were 3,482 DEGs (log2(Foldchange) > 1, *p* < 0.05), as shown in Figure 2A, The data has been submitted to the NCBI SRA database (Accession to cite for these SRA data: PRJNA916648, Temporary Submission ID: SUB12485493). In order to more intuitively display the number of genes in different FPKM intervals of each sample, we made statistics on the number of genes in three cases of FPKM in each group (FPKM ≤ 1, FPKM 1–10, FPKM ≥ 10), as shown in Figure 2B. A volcano plot in Figure 2C summarizes the gene expression differences (FDR) among control group, model group and experimental group. After clustering, the intersection of three groups of differential genes was obtained and a total of 125 genes were obtained, as shown in Figure 2D. Sequences longer than 200 bp were selected for Blastx homology alignment against GenBank to obtain accurate annotation information. According to the FPKM value, the expression values of 125 genes were visualized in the heatmap, as shown in Figure 2E. According to the MNC algorithm, the top 10 nodes were *CSF1, AREG, DDIT3, MAPK9, MAP3K7, ETV5, AKT3,* and *CCNA1*, and the genes related to autophagy were *CSF1, AREG, MAPK9, MAP3K7,* and *AKT3*.

### 3.3 GO and KEGG enrichment analyses of differentially expressed genes

**FIGURE 3:**
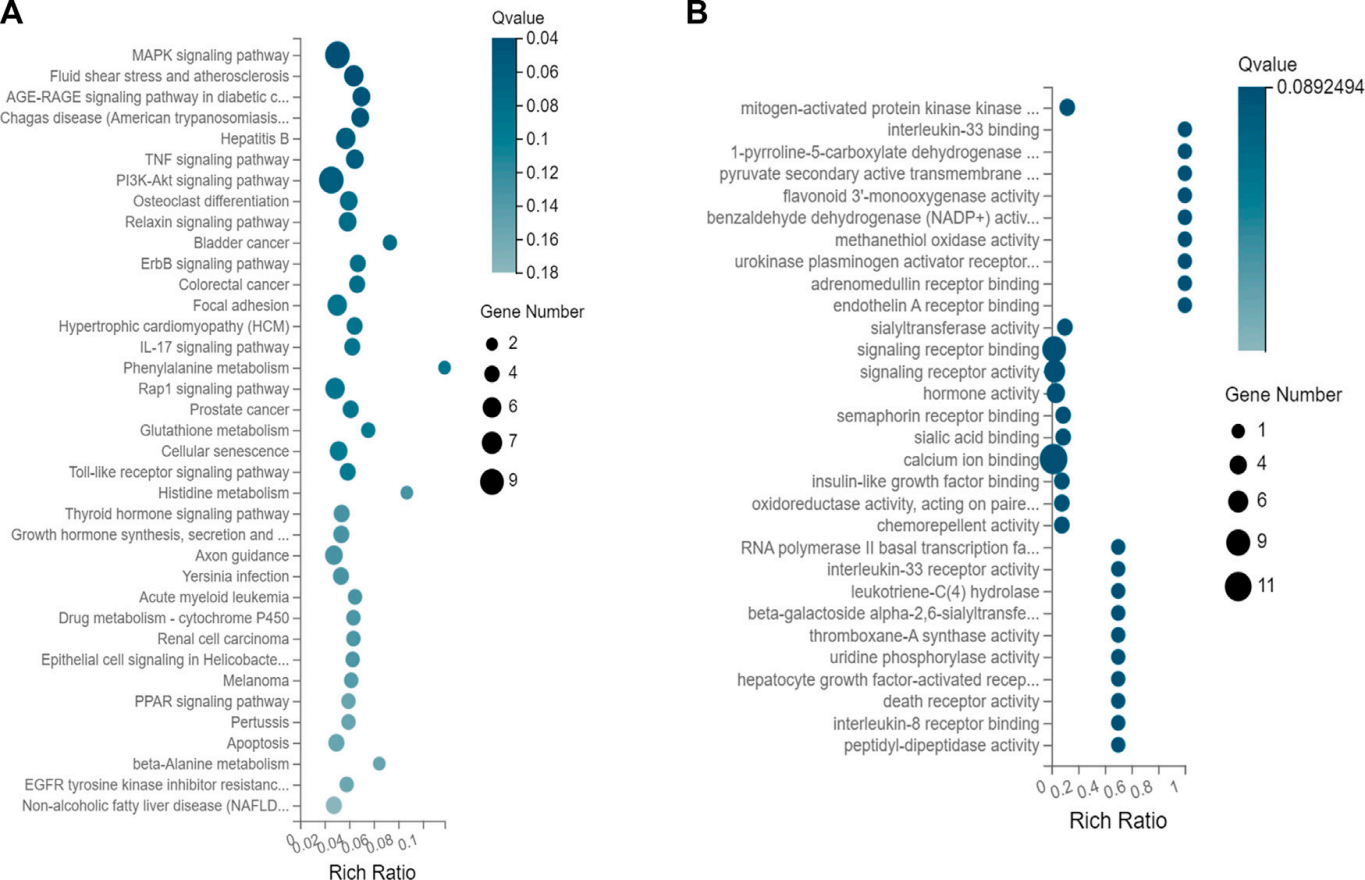
**(A)** KEGG pathway enrichment of DEGs. **(B)** Enrichment of target genes for GO biological processes.

The results of a KEGG pathway analysis suggest that DEGs were mainly related to the MAPK signaling pathway, as shown in Figure 3A. Terms in the GO biological process category were mainly related to mitogen-activated protein kinase binding (GO:0031434), interleukin-33 binding (GO:0002113), 1-pyrroline-5-carboxylate dehydrogenase activity (GO:0003842), and pyruvate secondary activity transporter activity (GO:0005477), as shown in Figure 3B.

### 3.4 qRT-PCR verified that Xuanfei Pingchuan capsule down-regulated the expression of autophagy-related genes.

**FIGURE 4:**
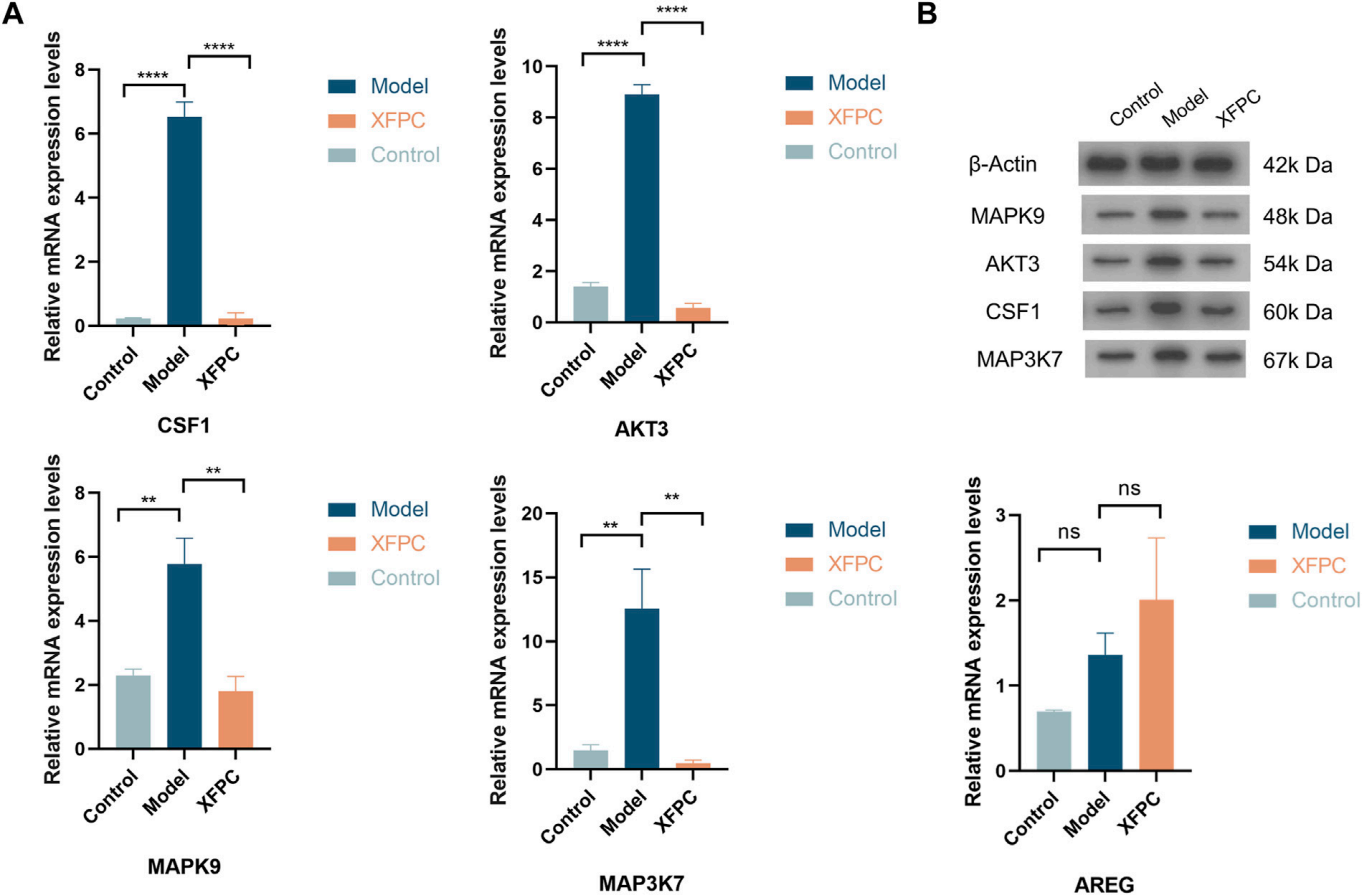
**(A)** mRNA expression levels of autophagy-related genes in XFPC and model groups; ^****^ *p* < 0.001, ^**^ *p* < 0.05. **(B)** Band pattern of autophagy-related proteins.

The autophagy-related genes *CSF1, MAPK9, MAP3K7, AKT3*, and *AREG* were selected for verification by qRT-PCR. The values of log2 (fold change) of *CSF1, MAPK9, MAP3K7*, and *AKT3* were significantly lower in the XFPC group than in the model group (*p* < 0.05). These results indicated that XFPC capsules down-regulated the expression of autophagy-related genes -- *CSF1*, *MAPK9*, *MAP3K7* and *AKT3*, consistent with the transcriptome sequencing results. There were no significant differences in *AREG* levels among groups, as shown in Figure 4A.

### 3.5 Western-blot verified that Xuanfei Pingchuan capsule down-regulated the expression of autophagy-related protein.

The autophagy-related proteins *CSF1*, *MAPK9*, *MAP3K7*, *AKT3*, and *AREG* were selected for verification by western blotting. The expression levels of *CSF1*, *MAPK9*, *MAP3K7*, and *AKT3* in the model group were significantly higher than those in the control group (*p* < 0.01). The expression levels of histone in the XFPC capsule group were significantly lower and *AKT3* levels were highly significantly lower than those in the control group (*p* < 0.01). These findings suggest that XFPC capsules down-regulated the expression of autophagy-related proteins, consistent with the transcriptome sequencing results, as shown in Figure 4B.

## 4 Discussion

The social burden of COPD is increasing year by year; in the United States, the cost of treating COPD is expected to reach $800.09 billion over 20 years or about $40 billion per year (Yin et al., 2016). Nearly 50% of patients with COPD show symptoms of airway mucus hypersecretion (chronic cough and sputum production) (Clémence Martin et al., 2019); mucus oversecretion results in increased mucus viscosity; additionally, cilia become shorter, the lodging, shedding, and ciliary wiggling ability is weakened, and the mucus removal ability is reduced. Therefore, the ability to remove foreign bodies, pathogenic bacteria, and other substances is reduced, resulting in continuous inflammation and obstruction and remodeling of the airway and causing repeated respiratory tract infections. Mucin secretion is regulated by a variety of signaling pathways, and the mitogen-activated protein kinase (MAPK) phosphorylation cascade plays an important role in regulating the expression of *MUC5AC*. As a member of the MAPK family, p38 MAPK can play an important role in the acute exacerbation of COPD by activating key transcription factors and inducing increases in the release of various cytokines, as confirmed by our previous research (Xue et al., 2021).

Long-term chronic exposure to smoking and environmental factors induces adaptive changes and apoptosis in bronchial epithelial cells, involving autophagy. The process of autophagy is mainly divided into the following stages: the formation and extension of autophagosomes, the fusion of autophagosomes and lysosomes, and degradation (Guo et al., 2017). Studies have found that the degree of shortening of cilia in airway epithelial cells and lung tissue is related to the degree of increase in autophagy levels (Wang Huanan et al., 2018). CSE can cause a decreased ciliary length or even the death of airway epithelial cells, which are replaced by goblet cells, resulting in the secretion of a large amount of mucus, alterations in cilia function, and airway mucus hypersecretion (AMH) (Petit Aureliel et al., 2019; Lv Xiaoxi et al., 2020). Increased autophagic bodies and autophagic lysosomes have been detected in the lung tissue of a rat model of COPD, and these contained a large number of cilia and related proteins; when an autophagy gene was silenced, neither the airway epithelial cells nor the cilia in lung tissue were shortened (Zhou Jie-Sen et al., 2016). COPD is clearly associated with a high level of autophagy, resulting in the loss of cilia and affecting ciliary motor function, suggesting that autophagy is an important factor in the aggravation of AMH(Wang et al., 2017). Therefore, the level of autophagy is closely related to the function of cilia in the lungs as well as the inflammatory response, and ciliary dysfunction and intensification of the inflammatory response are important factors leading to COPD-induced AMH. However, the specific mechanism linking autophagy and COPD-induced AMH has not been fully elucidated and requires further in-depth research.

XFPC capsules are a hospital preparation developed at the Shanxi Academy of Traditional Chinese Medicine (Shanxi Provincial Hospital of Traditional Chinese Medicine). It is developed by Professor Wang Xixing, a master of traditional Chinese medicine, who has continuously adjusted and improved the formula based on many years of clinical experience. Since 2005, it has been used in clinical applications (trade name: XuanFeiPingChuan capsule, preparation number AZ20080272), with an average annual use of more than 80,000 boxes. It is composed of 16 traditional Chinese medicines, including roasted ephedra, angelica, platycodon grandiflorum, almond, Su Zi, pinellia ternata, aster, winter flower, scapula seed, white mulberry, scutellaria baicalensis, schisandra chinensis, ginkgo, gecko, astragalus and licorice, and is based on the treatment principle of facilitating the flow of gastric qi to relieve asthma, clear heat, and eliminate sputum. From the perspective of traditional Chinese medicine (TCM), cellular autophagy is a dynamic self-balancing process that influences various aspects of the pathogenesis of COPD and is a manifestation of the lung-dispersing function, which is a crucial mechanism by which the body transforms and gets rid of various substances, e.g., endogenous phlegm and stagnant blood (Wang et al., 2021). Chinese medicine can enhance cellular autophagy and the “self-digestion” of protein fragments and subcellular organelles that accumulate, thus achieving a qi-blood balance. Although it is still controversial to study the molecular mechanism of autophagy in the prevention and treatment of COPD from the perspective of the qi-blood balance, and the function of TCM in regulating autophagy of COPD cells by promoting lung and relieving asthma. The early clinical research of the research group showed that XFPC capsules significantly improved AMH symptoms such as cough and expectoration in COPD patients compared with modern medicine treatment control group (Wang et al., 2016; Xue et al., 2012). *In vivo* experiments confirmed that XFPC capsules could significantly express inflammatory factor TNF-α,IL-8,IL-1β in serum of COPD rats, and promote the repair of damaged lung tissue compared with Dexamethasone treatment control group (Xue et al., 2016a; Xue et al., 2016b; Xue et al., 2017). It is confirmed that XFPC capsules can achieve safer and longer curative effect than symptomatic treatment in modern medicine. In the later stage, in order to further study the specific mechanism of XFPC capsules, the positive drug group was not set up. It is speculated that XFPC capsules may reduce airway inflammation by inhibiting autophagy level, and then AMH expression can express inflammatory factors in lung tissue. *In vitro* experiments, it was found that XFPC capsules could induce the expression of microtubule-associated protein LC3 in HBE induced by CSE. The key factors of AMH, MUC5AC and inflammatory factors suggest that XFPC capsules may inhibit the formation of AMH in COPD by regulating autophagy and inflammation (Xue et al., 2021).

In modern medicine, autophagy in COPD is a complex process involving many gene regulation. In this study, a transcriptome analysis was performed to evaluate the effects of XFPC capsules on CSE-induced HBE. The original image data obtained by sequencing is converted into raw sequence data (raw data or raw reads) through base calling, and stored in the format of fastq (fq) file, which contains the sequence of reads and sequencing quality information. A total of 16,507 genes were detected, and 3,482 differentially expressed genes were obtained. In the model group, 344 genes were up-regulated and 291 genes were down-regulated; The experimental group down-regulated 1,369 genes and up-regulated 724 genes. According to the results of differential gene detection, the R packet pheatmap is used for hierarchical clustering analysis of the union differential genes. KEGG is the main public database with pathway information for genes (Kanehisa et al., 2021). The KEGG pathway analysis in this study suggested that it is mainly related to MAPK signaling pathway, Fluid shear stress and spheroclysis, AGE-RAGE signaling pathway in diabetic applications, *etc.* The GO function significance enrichment analysis gives the GO function items that are significantly enriched in the candidate genes compared with the whole gene background of the species, thus giving the biological functions of the candidate genes that are significantly related. GO biological processes in this study is mainly related to mitogen-activated protein kinase binding, interleukin-33 binding, and 1-pyrroline-5-carboxylic acid dehydrogenase activity, which are associated with genes related to autophagy.

This study verified the transcriptome results by qRT-PCR and western blotting, and confirmed that the autophagy-related genes *CSF1, MAPK9, MAP3K7,* and *AKT3* were significantly upregulated in the model group and downregulated in the experimental group. Collectively, these results indicated that XFPC capsules downregulate the expression of autophagy-related genes. Colony-stimulating factor 1 (*CSF1*) is a secreted cytokine that causes receptor dimerization by binding with receptor CSF1R, and then activates CSF1R and causes partial intracellular autophosphorylation of the receptor (Renda et al., 2008; Rovida et al., 2008). Similar to other tyrosine kinase receptors, the biological activity of CSF1R is primarily mediated by three signal transduction pathways: LPC-γ, non-receptor tyrosine kinase (JAK-STAT), and MAPK(Wen et al., 2023). CSF1/CSF-1 promotes the expression and phosphorylation of ULK1, thereby inducing autophagy (Yang et al., 2020). *AKT3* is the least researched of among the three closely related serine/threonine protein kinases (*AKT1*, *AKT2*, and *AKT3*) involved in metabolism, proliferation, cell survival, growth, and angiogenesis (Zhang et al., 2022). Activating AKT3/MTOR conduction can induce autophagy flux impairment and cell apoptosis (Ouyang et al., 2022). Mitogen-activated protein kinase nine is encoded by the *MAPK9* (*jnk2*) gene in humans, which together with *MAPK8* (jnk1) and *MAPK10* (jnk3) form the c-Jun amino-terminal kinase (JNK); the JNK signaling pathway is an important branch of the *MAPK* pathway. The human *MAP3K7* gene encodes mitogen-activated protein kinase 7, also known as *AKT1*, which plays a significant role in the MAPK signaling pathway. *MAP3K7* mutations cause the dysregulation of downstream TAK1-dependent signaling pathways and decreased autophosphorylation. Loss The loss of *TAK1* function is associated with impaired transforming growth factor-β (TGF-β)-mediated *a* smooth muscle actin (α-SMA) cytoskeleton assembly and cell migration as well as defective autophagic processes (Micale et al., 2020). So CSF1,MAPK9, MAP3K7 and AKT3 are related to autophagy, but there is no evidence that these genes are involved in the pathology of AMH, this will be a new research direction.

TAK1-JNK/p38 is composed of TAK1 and its downstream c-jun N-terminal kinase or stress-activated protein kinase (JNK) and p38. Previous studies have shown that activated JNK/p38 can participate in promoting airway epithelial cell chemokines to stimulate immune cell inflammatory factors to participate in the occurrence of chronic inflammation in COPD(Liu et al., 2021). Activated JNK can cause AP-1 activation and start MUC5AC gene transcription. CSE can induce the activation of JNK and c-Jun in HBE cells and mice and significantly increase the expression of IL-6 and MUC5AC. The expression of MUC5AC in HBE cells after inhibiting the phosphorylation of JNK and p38 (Wu et al., 2021). *MAP3K7* activates mitogen-activated protein kinase 4 (*MKK4*) and mitogen-activated protein kinase 7 (*MKK7*) by activating TAK1, which in turn phosphorylates and activates JNK/p38 pathway. Based on this, we speculate that Xuanfei Pingchuan Fang can reduce autophagy by inhibiting the TAK1-JNK/p38 pathway, so as to reduce inflammation and thus alleviate the expression of AMH caused by COPD.

## 5 Conclusion

Our results indicated that XFPC capsules could reduce autophagy levels, maintain ciliary function, and improve the inflammatory status *in vivo* by interfering with the expression of *CSF1*, *MAPK9*, *MAP3K7*, and *AKT3* in the MAPK pathway suggesting that TAK1-JNK/p38 pathway may be the target pathway of Xuanfei Pingchuan Fang in treating COPD. These findings explain the molecular mechanism underlying the therapeutic effects of XFPC capsules in COPD-induced AMH, provide novel therapeutic targets, and offer a scientific foundation for the development effective treatments for COPD, which has important implications for lessening the burden on patients and society.

## Data Availability

The datasets presented in this study can be found in online repositories. The names of the repository/repositories and accession number(s) can be found in the article/supplementary material.
